# Painful Diabetic Neuropathy in Japanese Diabetic Patients Is Common but Underrecognized

**DOI:** 10.1155/2013/318352

**Published:** 2013-11-26

**Authors:** Mayumi Tsuji, Tetsuyuki Yasuda, Hideaki Kaneto, Taka-aki Matsuoka, Takahisa Hirose, Ryuzo Kawamori, Masako Iseki, Iichiro Shimomura, Masahiko Shibata

**Affiliations:** ^1^Department of Metabolic Medicine, Osaka University Graduate School of Medicine, 2-2 Yamadaoka, Suita, Osaka 565-0871, Japan; ^2^Division of Diabetes, Metabolism, and Endocrinology, Department of Medicine, Toho University School of Medicine, 5-21-16 Ohmorinishi, Ota-ku, Tokyo 143-8540, Japan; ^3^Sportology Center, Juntendo University Graduate School of Medicine, 3-1-3 Hongo, Bunkyo-ku, Tokyo 113-8431, Japan; ^4^Department of Anesthesiology and Pain Management, Juntendo University School of Medicine, 3-1-3 Hongo, Bunkyo-ku, Tokyo 113-8431, Japan; ^5^Pain Medicine, Osaka University Graduate School of Medicine, 2-2 Yamadaoka, Suita, Osaka 565-0871, Japan

## Abstract

Although chronic pain due to diabetic neuropathy, defined as painful diabetic neuropathy (PDN), is a debilitating and distressing complication of diabetes, epidemiological data on PDN has been scarce, especially in Asia. We evaluated the prevalence of Japanese PDN and its impact on their quality of life (QOL) and metnal state. In addition, we examined to which extent physicians are aware of patients' PDN. A total of 298 patients with diabetes were found to be eligible for the study. We revealed that substantial percentage (22.1%) of Japanese diabetic patients had PDN and that PDN had negative effect on patients' QOL and mental state. However, physicians were aware of PDN in only 36.4% of patients with the condition. To the best of our knowledge, this is the first report showing the extent of physicians' awareness of patients' PDN. In conclusion, physicians treating diabetes need to be more aware of patients' PDN in everyday clinical practice to prevent the progression of PDN and improve the patients' QOL and mental state.

## 1. Introduction

Diabetic peripheral neuropathy is one of the most common long-term complications in diabetic patients and is the main initiating factor for foot ulceration and lower limb amputation [1]. In addition, diabetic peripheral neuropathy is associated not only with significant reduction in quality of life (QOL) but also with increased mortality [2]. The prevalence of diabetic peripheral neuropathy is 47% of all diabetic patients when nerve conduction testing is used for diagnosis [3]. Patients with diabetic peripheral neuropathy often experience chronic pain defined as painful diabetic neuropathy (PDN), which starts in both feet and often leads to involving calves, fingers, and hands (glove and stocking pattern). The symptoms are typically characterized as prickling, burning, and like an electric shock with nocturnal exacerbations. PDN not only causes pain, but also has negative impact on patients' sleep, mood, mental state, and daily activities resulting in poor QOL [4, 5]. Thus, PDN is a debilitating and distressing complication of diabetes. However, unlike other diabetic vascular complications including retinopathy, nephropathy, and atherosclerosis, PDN has not been extensively studied, and its epidemiological data has been scarce, especially in Asia. In addition, there have been few reports examining how diabetic physicians are aware of patients' PDN. In this study, we evaluated the prevalence of Japanese diabetic patients with PDN and its impact on their QOL and metnal state. In addition, we examined to which extent physicians are aware of patients' PDN.

## 2. Patients and Methods

Patients were recruited from outpatients who attended Osaka University Hospital or 17 diabetes clinics in Osaka, Hyogo, and Tokyo for treating diabetes from 2010 to 2012. Eligibility was limited to adult diabetic patients (≥18 years) with more than 5-year durations of diabetes and treatment with antidiabetic agent. We excluded patients who had neurologic disorders and other pain conditions unrelated to diabetic peripheral neuropathy, diabetic gangrene, peripheral artery disease, spine and psychological disorders, malignancy, or alcohol abuse or alcoholism. Patients completed questionnaire about their experience of chronic pain in bilateral legs or arms, quality of life, and mental state. The presence or absence of patients' painful symptoms was confirmed by the following question: “Do you have chronic painful or discomfort symptoms in bilateral legs or arms?” QOL was measured by the Short-Form 36-Item Health Survey Questionnaire (SF-36) consisting of 36 questions yielding eight different subscales. A score from 0 to 100 (worst to best) was calculated for each subscale [6]. Mental state was assessed through the Hospital Anxiety and Depression Scale (HADS), consisting of seven questions regarding anxiety (HADS-A) and depression (HADS-D). A score from 0 to 21 (best to worst) was calculated for each subscale [7]. This study was approved by the local research ethics committee and all participants gave written informed consent. Statistical analyses were performed using Stat-View statistical software (Version. 5.0 for Windows; Abacus Concepts, Berkeley, CA, USA). Continuous variables are expressed as means ± SD. Comparisons between groups were made with the  *χ* ^2^  test or Student's  *t*-test as appropriate. A two-sided value of  *P* < 0.05  was considered statistically significant.

## 3. Results

A total of 298 patients with diabetes (male : female 176 : 122) were found to be eligible for the study. Patients' characteristics in the study were as follows: age,  61.1 ± 10.4  years old; duration of diabetes,  13.4 ± 6.9  years; body mass index,  24.5 ± 4.5 kg/m^2^;  HbA_1c_,  7.4 ± 1.2%; systolic blood pressure,  129.3 ± 46.5 mmHg; diastolic blood pressure,  73.5 ± 12.7 mmHg; Cr,  0.82 ± 0.65 mg/dL; triglyceride,  144.3 ± 107.0 mg/dL; LDL cholesterol,  110.3 ± 29.6 mg/dL; HDL cholesterol,  57.7 ± 18.4 mg/dL; and current smoking, 17.1%. The prevalence of patients with PDN was 22.1%. The abovementioned clinical parameters were not statistically different between the patients with and without PDN (Table 1). It is noted that in only 36.4% of patients with PDN, their PDN was recognized by physicians. In the SF-36, all subscale scores except for mental health were significantly lower in the patients with PDN than those without PDN. HADS-D scores were significantly higher in the patients with PDN than those without PDN. HADS-A scores were also higher in the patients with PDN than those without PDN, although it did not reach a statistical significance (Table 1).

## 4. Discussion

In this study, we demonstrated that substantial percentage (22.1%) of Japanese diabetic patients had PDN. In addition, patients with PDN had poorer QOL and more depressive state compared with those without PDN. However, physicians were aware of only 36.4% of patients' PDN. To the best of our knowledge, this is the first report showing the extent of physicians' awareness of patients' PDN.

The prevalence of diabetic patients with PDN in the present study is similar to that reported in recent studies in Europe; the prevalence of PDN was 14–26% [8–11]. The study with 350 diabetic patients by Daousi et al. in United Kingdom (UK) showed that the prevalence of PDN was 16%. On the other hand, the prevalence of chronic neuropathic pain in an age- and gender-matched nondiabetic population was 5% [8]. The study with 269 type 2 diabetic patients by Davies et al. in UK showed that the prevalence of PDN was 26% [9]. The study with 1111 diabetic patients by van Acker et al. in Belgium showed that 14% of the patients had lower limb neuropathic pain [10]. Above different prevalence of PDN including our study may be partially explained by different design and inclusion criteria for PDN. Taking these reports including our study into consideration, the prevalence of PDN in diabetic patients would be estimated at around 20%. Previous studies have revealed that PDN has negative effect on QOL and metnal state [5, 9, 10]. This study confirms a statistically significant negative effect of PDN on QOL and depressive state. However, despite high prevalence and negative impact of PDN, physicians' awareness of PDN in the present study was poor. There have been few studies evaluating the discrepancy between the patients' complain and physicians' awareness of patients' symptom in the field of pain. Although its detailed reasons are unclear, we can assume several reasons. First, patients may not complain of painful symptoms to physicians. Actually, in a study of 350 diabetic patients in UK, 12.5% of patients with PDN had never reported their symptoms to physicians [8]. Although there is no apparent evidence, we assume that the relationship between the patients and physicians may be not good enough for some patients to complain symptoms easily to physicians, or that Japanese patients may have higher tolerance for pain than other ethnics or consider their tolerance for pain as virtue. Second, physicians may not ask patients about painful symptoms because of the lack of effective therapy for PDN. Actually, in Japan, effectual drugs for PDN such as pregabalin, duloxetine, tricyclic antidepressant, or opioid were not commonly used during the present study because of the lack of indication of health insurance for these drugs. However, because the above reasons are our hypotheses, we should clarify the reasons of discrepancy between the prevalence of patients' painful symptoms and physicians' awareness of patients' symptom in the future study. It has been reported that HbA_1c_, duration of diabetes, hypertension, and smoking are risk factors for diabetic peripheral neuropathy [12]. However, there was no difference in these clinical factors between the patients with and without PDN in the present study. Similar to our results, it was reported that there was no difference in age, sex, duration of diabetes, body mass index, and glycemic control between the patients with and without PDN [7]. Therefore, at this time, because risk factors for PDN remain unknown, we think that physicians should treat diabetic patients in clinical practice with the recognition that risk factors for PDN remain unknown until they are revealed in further large studies. There are several limitations in this study. First, this study is a patient-reported questionnaire survey with relatively small number of patients. Second, this study was conducted by limited Japanese diabetic physicians. Therefore, this study has selection and recall bias, and consequently, the results and conclusion reported here might not be generalized to other populations.

## 5. Conclusions

Although PDN is a common complication leading to negative effect on QOL and depressive state, physicians' recognition is poor. Physicians treating diabetes need to be more aware of patients' PDN in everyday clinical practice to prevent the progression of PDN and improve the patients' QOL and mental state.

## Figures and Tables

**Table 1:** Clinical characteristics, SF-36 and HADS scores of the patients with and without painful diabetic neuropathy.

Parameters	Patients		*P *
	Without PDN	With PDN	
Number	232	66	
Gender (male/female)	143/89	33/33	0.12
Age (years)	60.7 ± 10.6	62.5 ± 9.8	0.24
Duration of diabetes (years)	13.2 ± 7	14.2 ± 6.6	0.35
BMI (kg/m^2^)	24.2 ± 4.5	25.4 ± 4.1	0.06
HbA_1c_ (%)	7.4 ± 1.1	7.6 ± 1.2	0.14
Hypertension (%)	56.5	53.3	1.0
Dyslipidemia (%)	70.3	72.7	0.76
Current smoking (%)	17.6	15.6	0.84
SF-36			
Physical function	47.7 ± 16.7	39.7 ± 16	<0.0001
Role physical	48.4 ± 16.6	43.6 ± 12.3	<0.005
Bodily pain	53.6 ± 15.9	46.9 ± 10.2	<0.0001
General health	47.2 ± 13.7	42.9 ± 9.7	<0.005
Social function	51 ± 14.4	47.4 ± 11.6	<0.05
Vitality	52.4 ± 15.3	48.8 ± 10.8	<0.05
Role emotional	49.8 ± 16	45.4 ± 11.1	<0.005
Mental health	52.3 ± 15.1	49.7 ± 10.2	0.06
HADS			
HADS-A	3.9 ± 3.1	4.7 ± 3.9	0.066
HADS-D	3.8 ± 2.9	5.2 ± 3.7	<0.005

Data are means ± SD. BMI: body mass index; SF-36: the Short-Form 36-Item Health Survey Questionnaire; HADS-A: the Hospital Anxiety and Depression Scale-Anxiety; HADS-D: the Hospital Anxiety and Depression Scale-Depression; PDN: painful diabetic neuropathy.

## References

[1] Boulton AJ, Vileikyte L, Ragnarson-Tennvall G, Apelqvist J (2005). The global burden of diabetic foot disease. *Lancet*.

[2] Hsu WC, Chiu SYH, Yen AMF (2012). Somatic neuropathy is an independent predictor of all- and diabetes-related mortality in type 2 diabetic patients: a population-based 5-year follow-up study (KCIS No. 29). *European Journal of Neurology*.

[3] Dyck PJ, Kratz KM, Karnes JL (1993). The prevalence by staged severity of various types of diabetic neuropathy, retinopathy, and nephropathy in a population-based cohort: the Rochester Diabetic Neuropathy Study. *Neurology*.

[4] Benbow SJ, Wallymahmed ME, Macfarlane IA (1998). Diabetic peripheral neuropathy and quality of life. *Monthly Journal of the Association of Physicians*.

[5] Galer BS, Gianas A, Jensen MP (2000). Painful diabetic polyneuropathy: epidemiology, pain description, and quality of life. *Diabetes Research and Clinical Practice*.

[6] Ware JE, Sherbourne CD (1992). The MOS 36-item short-form health survey (SF-36). I. Conceptual framework and item selection. *Medical Care*.

[7] Zigmond AS, Snaith RP (1983). The hospital anxiety and depression scale. *Acta Psychiatrica Scandinavica*.

[8] Daousi C, MacFarlane IA, Woodward A, Nurmikkot TJ, Bundred PE, Benbow SJ (2004). Chronic painful peripheral neuropathy in an urban community: a controlled comparison of people with and without diabetes. *Diabetic Medicine*.

[9] Davies M, Brophy S, Williams R, Taylor A (2006). The prevalence, severity, and impact of painful diabetic peripheral neuropathy in type 2 diabetes. *Diabetes Care*.

[10] van Acker K, Bouhassira D, de Bacquer D (2009). Prevalence and impact on quality of life of peripheral neuropathy with or without neuropathic pain in type 1 and type 2 diabetic patients attending hospital outpatients clinics. *Diabetes and Metabolism*.

[11] Erbas T, Ertas M, Yucel A, Keskinaslan A, Senocak M (2011). Prevalence of peripheral neuropathy and painful peripheral neuropathy in Turkish diabetic patients. *Journal of Clinical Neurophysiology*.

[12] Tesfaye S, Chaturvedi N, Eaton SEM (2005). Vascular risk factors and diabetic neuropathy. *New England Journal of Medicine*.

